# Designing an App to Overcome Language Barriers in the Delivery of Emergency Medical Services: Participatory Development Process

**DOI:** 10.2196/21586

**Published:** 2021-04-14

**Authors:** Eva Maria Noack, Jennifer Schulze, Frank Müller

**Affiliations:** 1 Department of General Practice University Medical Center Göttingen Göttingen Germany

**Keywords:** paramedic, interpreter, medical translation, application software, app, digital communication tool, foreign-language patients, language barrier, participatory design, prehospital emergency care, emergency medical service

## Abstract

**Background:**

In emergencies, language barriers may have dangerous consequences for the patients. There have been some technical approaches to overcome language barriers in medical care but not yet in the prehospital emergency care setting. The use of digital technologies in health care is expanding rapidly. Involving end users at all stages of the development process may help to ensure such technologies are usable and can be implemented.

**Objective:**

We aimed to develop a digital communication tool that addresses paramedic needs in the specific circumstances of prehospital emergency care and helps paramedics to overcome language barriers when providing care to foreign-language patients.

**Methods:**

We actively engaged paramedics and software designers in an action-oriented, participatory, iterative development process, which included field observations, workshops, background conversations, questionnaires on rescue missions, studying the literature, and preliminary testing in the field.

**Results:**

With input from paramedics, we created an app with 600 fixed phrases supporting 18 languages. The app includes medical history–taking questions, phrases asking for consent, and phrases providing specific additional information. Children as patients, as well as their carers and other third parties, can be addressed with appropriate wording. All phrases can be played back audibly or displayed as text. The comprehensive content is grouped into categories and adapted to diverse scenarios, which makes the tool rapidly usable. The app includes a function to document patient responses and the conversation history. For evaluation in a clinical study, the app is run on a smartphone with extra speakers to be of use in noisy environments. The use of prototypes proved valuable to verify that the content, structure, and functions discussed in theory were of value and genuinely needed in practice and that the various device control elements were intuitive.

**Conclusions:**

The nature of the paramedic work environment places specific demands on the communication options used and need for such devices. The active involvement of paramedics in the development process allowed us to understand and subsequently consider their experience-based knowledge. Software designers could understand the paramedics’ work environment and consider respective needs in the menu navigation and design principles of the app. We argue that the development of any medical software product should actively involve both end users and developers in all phases of the development process. Providing the users with the opportunity to influence technology development ensures that the result is closer to their needs, which can be seen as crucial for successful implementation and sustainable use.

**Trial Registration:**

German Clinical Trials Register DRKS00016719; https://www.drks.de/drks_web/navigate.do?navigationId=trial.HTML&TRIAL_ID=DRKS00016719

**International Registered Report Identifier (IRRID):**

RR2-10.1186/s12913-020-05098-5

## Introduction

For medical treatment, it is essential that patients and medical staff can communicate with each other. Language barriers can have a negative impact on the quality of care and patient outcomes [1,2]. Particularly, in out-of-hospital emergency situations that require rapid assessments and decision making and frequently necessitate initial treatment on the scene, language barriers can have dangerous consequences for patients [3,4]. In a more rural area of Germany, 2.2% of the rescue service operations involved patients with a language barrier [5]. This number is expected to be significantly higher in urban areas. In emergencies, it is therefore crucial for the paramedic staff to understand the patients’ acute complaints, pre-existing conditions, allergies, or drug treatments. However, at the emergency scene, accurate translation by professional interpreters is rarely available. Therefore, paramedics mostly rely on nonverbal communication, use a third language, or rely on lay interpreters [6,7]. Translation accuracy cannot be guaranteed when making use of interpretation support from bystanders, and confidentiality problems arise [1,2].

There are several technical solutions to improve patient-staff communication in multilingual clinical settings. In hospital and primary care, video or telephone interpreting services may be used [8-10]. For time-critical emergencies on potentially dangerous sites, these are often not available and would require a reliable network coverage, which is rarely guaranteed in rural areas in Germany. The quality of machine translation such as Google Translate technology is improving, but it is still not considered accurate enough for actual deployment in health settings [11,12], and similarly, ad-hoc translators require a permanent connection to the internet. In addition to reliability issues, data confidentiality problems are unsolved.

Communication tools to overcome language barriers in health care must be developed and suitably adapted to the specific working environment and health care setting. Examples include a tablet-based app for medical history taking for refugees and asylum seekers in a German transit camp, which was developed and piloted in a family doctor surgery setting [13,14]; a speech-enabled, fixed-phrase translator was found to be a good alternative to collecting information if interpreters are not available in an emergency room setting [15]. However, these tools do not suit prehospital emergency care situations, which are frequently complex, volatile, and rushed. A tool for rescue operations therefore requires not only adapted content but also a different communication approach.

In the project “DICTUM rescue” (Digital cross-cultural interpreter-tool in medical consultations for refugees and migrants), we aim to develop a digital communication tool that helps paramedics to overcome language barriers when providing care to foreign-language patients in the prehospital emergency care setting.

Digital technologies have the potential to simplify work processes and improve productivity. However, if such tools do not meet user needs or are inconvenient to use, they will be used reluctantly or not at all. Involving end users may make digital innovations more suitable for their use, which will consequently improve technology uptake. While there are examples of patients, medical professionals, and other stakeholders being successfully involved in the design of medical technology [16-18], they rarely take part in the development process.

By involving paramedics and software designers in the development, we aimed to develop an app that meets the needs of paramedics when providing care to foreign-language patients and accommodates the specific circumstances of rescue operations. In this paper, we focus on the app development process and paramedics’ active involvement in this process.

The app will be evaluated in an interventional trial (No. DRKS00016719) [19]. 

## Methods

### Action-Oriented Participatory Approach: Designing for, With, and by the Users

We used an action-oriented participatory approach, actively involving paramedics from 4 emergency medical service stations in Lower Saxony, Germany. Action-oriented approaches are devoted to defining problems and finding respective solutions in real-world situations together with the people who experience them [20,21]. In the development and implementation of health technologies, participatory designs have been used and perceived as helpful [22,23]. Our approach combined aspects that can be found in “classical” participatory research as well as in participatory design. While the first usually operates with clear, predefined research questions and endpoints, the latter is described as a process-based and rather open development, where an object, tool, or service is designed [24]. The idea behind our approach was: If this app is to succeed in improving paramedic communication with foreign-language patients, it has to meet the specific needs of paramedics and accommodate the particular setting of emergency medical services. Software designers (aidminutes GmbH) were similarly engaged in the participatory approach from the beginning so they could understand paramedic expectations and requirements for rescue missions. The research team consisted of 2 medical scientists and a study nurse, a trained paramedic. The development process and documentation should strictly follow scientific criteria and include external evidence, for example from literature, and meet ethical standards of qualitative research. For the realization of the project, we obtained ethical approval from the research ethics board of the University Medical Center Göttingen (Ethics Approval No. 9/9/18).

We used several techniques to investigate specific aspects for content and technical development (Table 1). An extensive literature search of relevant textbooks and guidelines accompanied the development process. Data were collected using records, field notes, and minutes.

**Table 1:** Overview of the techniques used for app design.

Timeframe	Activity	Participants	Aim	Data collection and analysis
Dec 2018 - Mar 2019	Field observations (“internship”)	Software designers	Experience paramedics’ daily work, gain knowledge regarding the nature of the role and its specific challenges.	Observations as to the requirements and possible challenges for the app were discussed within the team and with paramedics.
Feb 2019 - Sep 2019	Workshops	Paramedics from the rescue stations and software designers	Different foci and formats: Discuss and define content and structure; simulate cases in role play to evaluate content and structure using paper prototypes; identify useful functions; check hardware options.	Paramedics discussed content and structure and reflected on the role plays. Findings (minutes of discussions and suggestions, observations of simulations) were summarized by the research team, discussed, checked with literature and guidelines, and included in new versions. Findings as to functionality were discussed with software designers.
Jan 2019 - Dec 2019	Background conversations	Paramedics and relevant management (heads and deputies of the rescue stations, the district’s rescue service and fire department)	Provide a quick opinion and preliminary appraisal of a feature, wording or content; test app prototypes and assess perception; keep the staff informed about the progress of the project; give paramedics the ongoing opportunity to share their experience and thoughts with research team; plan implementation in daily routine.	Discussions were held within the research team and with software designers.
Feb 2019 - (ongoing)	Questionnaires	Paramedics	Gain information on real emergencies with foreign-language patients; enable paramedics to promptly describe their respective experience and impressions; detect which languages have to be supported by the app.	Descriptive statistics (scaled questions), frequency analysis (languages), content analyses (free texts); challenges raised could be reflected on the workshops.
Sep 2019 - Oct 2019	Case simulations	Students in the School for Paramedics	External perception of the developed app; test and evaluate the app in a mock real-life setting; finalize the app.	Observation of the simulations by the research team and software designers; students provide feedback on user experience.
Dec 2019	Final (“prerelease”) testing	Two paramedics from each rescue station	Test and evaluate the app in a real-life setting.	Direct feedback to developers and researchers.

**Figure 1:**
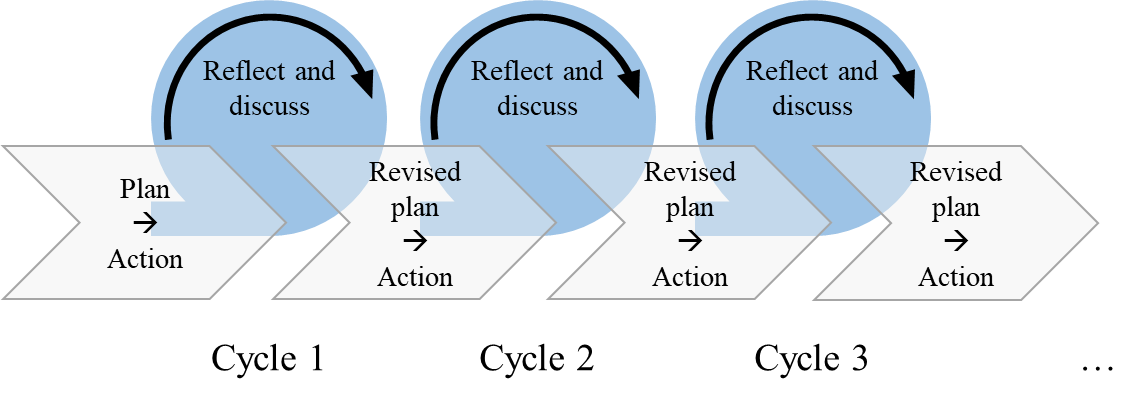
Iterative process.

The research and development process moved in iterative cycles (Figure 1). At each stage, findings from the previous cycle were reflected on and formed the basis for planning the next phase. For example, intermediate results concerning the communication content (which phrases should be integrated?) and its structure (how should these phrases be grouped?) were discussed and tested in role play. As new components arose, the structure was revised, the proposed content was adjusted to reflect up-to-date literature and guidelines, features were rethought with technical developers, and the features were set up for discussion and tested again. The number of iterations could not be predetermined beforehand as interesting outputs could not be anticipated and could arise at any stage.

### Workshops and Background Conversations

We invited the rescue station staff to participate in various workshops. These had different formats, including discussions and role plays, adapted to the shifting foci, for example the app’s content, structure, functions, appearance, device options, and implementation in daily routine. To test the app content and structure, we simulated rescue missions with foreign-language patients using paper prototypes presented in booklet format (see Multimedia Appendix 1).

We conducted 8 workshops with 4-8 participants each, which lasted 2-3 hours. We tried to ensure variation in participant work experience, age, and gender. In total, 47 paramedics participated in the workshops.

To obtain feedback on usability and appearance, we presented design mock-ups, click dummies, and finally alpha and beta versions of the app. We also encouraged workshop participants to verbalize their opinions and to “think aloud” while carrying out app-related tasks. This helped us to understand how paramedics experienced and assessed the user interface and to test whether they used control elements and found the desired phrases intuitively.

### Questionnaires on Rescue Missions With Foreign-Language Patients

During the app development phase and as part of the clinical trial, paramedics from the participating rescue stations completed questionnaires after they had provided care to foreign-language patients. These questionnaires provided us with information on how paramedics had experienced the communication with patients and how they had dealt with obstacles. This information complemented the findings from the other activities and could be included in the discussions with paramedics. The questionnaires also helped us to decide which languages should be supported by the app to meet local demand.

### Case Training Simulating Emergency Situations for External Testing

To make sure that the app could be used intuitively by paramedics who had not been involved in the development process, we tested early versions of the app with third-year and final-year paramedic students. At the school for paramedics, case simulations for training purposes are comprised of a complete set of emergency equipment for a rescue mission. During a simulation, the instructor can remotely control the devices, for example changing a patient’s vital parameters and thereby creating a more realistic working environment. We ran 16 case simulations including internal medicine, traumatology, gynecology, and pediatrics in 2 student groups consisting of 5 and 6 students, respectively. Scenarios were prepared by the instructor who also did not know the app. In each simulation, a team of 2 students treated a Dari-speaking patient. Afterwards, the simulations were discussed with the instructors and the students observing the simulation. The researchers and software designers additionally observed the interactions.

### Prerelease Testing of the App for Final Refinements and Implementation

At each rescue station, 2 paramedics tested the app and were asked to provide feedback to the technical developers and researchers for final refinements before implementation. The aim in this phase was to observe performance and the stability of the app in real rescue operations. We established a private messenger channel for direct feedback.

## Results

### Developing in Loops

**Figure 2:**
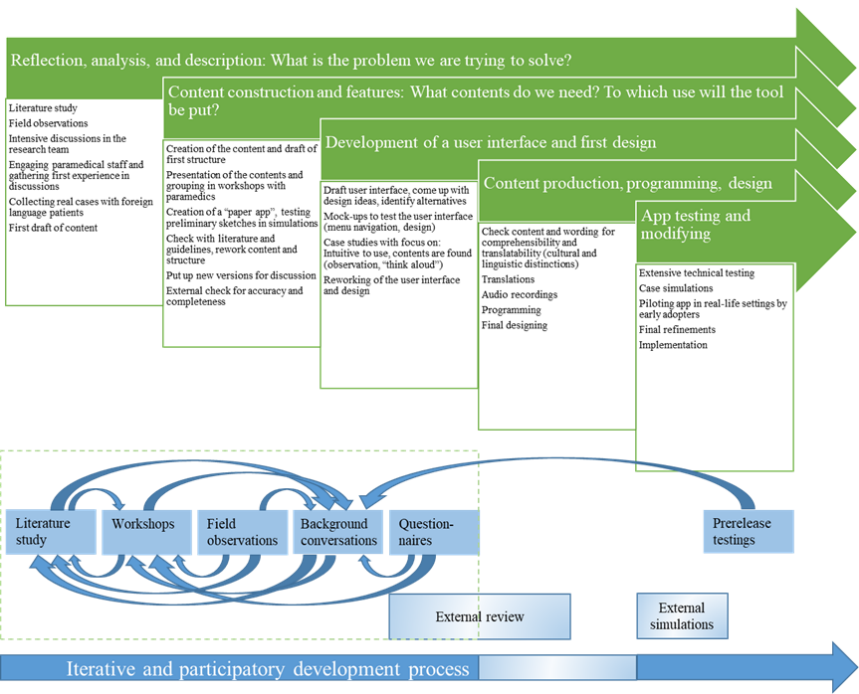
App development process.

We started with initial reflections on the field and on app principles. Subsequently, we collected and drafted questions and other phrases that reflected communication situations in emergency medical services. Concurrently, we set up a structure that allowed rapid and flexible medical history taking, adapted to a diverse array of scenarios. We defined the necessary functions that had to be considered in the design of the user interface, explored which devices best suited rescue missions, discussed how to implement the app in daily routine, and established which languages were needed to meet the local population. Finally, we conducted external app testing sessions. Figure 2 gives an oversight of the process of the app development and shows the main feedback loops.

### Initial Reflections

An app that helps overcome language barriers in prehospital emergency situations needs to be reliable, rapid-to-use, and usable offline and has to be run on a handy and sturdy device. The app operator should be the paramedic, as patients might not be able to use a digital device or maintain good hygiene.

To enable paramedic-patient communication that resembles as closely as possible a normal verbal interaction and to include illiterate and myopic patients, all phrases needed to be provided as audible messages. For app use in noisy surroundings, with hearing impaired patients, or to ensure confidential matters would be addressed sensitively and discreetly, phrases should also be displayed in text on the device.

Speech recognition is not yet reliable enough for clinical deployment, especially in situations where the environment is noisy, several speakers are involved, and colloquial expressions or dialects are being used. Therefore, all questions have to be posed in a way that patients can answer them nonverbally; that is, questions have to be closed-ended or contain a request to show something. Patients have to feel confident on how to answer, and the (nonverbal) answers have to be understood unambiguously by the paramedics. If it is not clear which language a patient understands, the patient should be able to select the language she or he prefers. In addition, it should be possible to check whether the patient understands the selected language.

**Figure 3:**
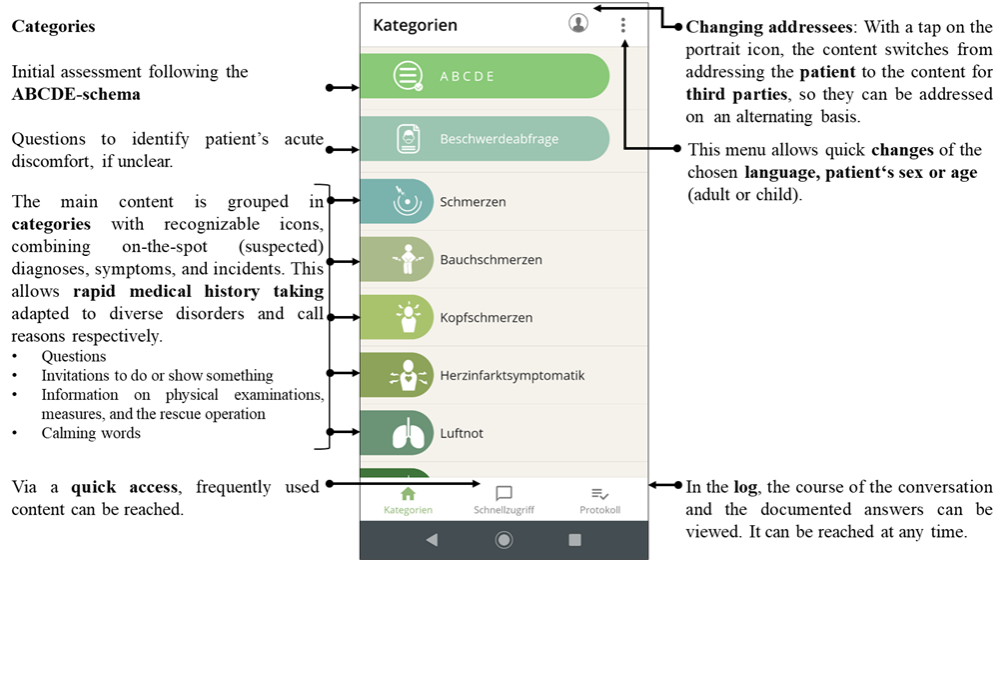
Main functions of the app and structure (categories; see text for further explanation).

The solution for our study consists of a smartphone-based app. The app’s main functions are outlined in Figure 3 and described in the following sections.

### Content

The aim of the first set of activities was to identify the key issues among paramedics in the participating emergency medical stations in the context of rescue missions with foreign-language patients and to determine the communication requirements in such missions.

Paramedics ask questions but they also give information and ask for consent, for example, when they perform measures or (drug) therapy on the patient. Communication also takes place with third parties, for example relatives or bystanders, and patients and third parties are often addressed on an alternating basis.

Pediatric treatment in emergency scenarios requires different content and wording. We therefore devised a child mode that covers children-specific emergencies and provides phrases that can be understood by children. Moreover, the content is tailored to the patient’s gender.

These requirements led to an unexpectedly high number of phrases needed, which was a challenge for structuring the content and for the user interface design.

Based on these initial insights, a first draft of questions and phrases was set up for discussion among the paramedics, which included determination of those questions that were indispensable for differential diagnoses, further treatment, the next steps, or for paramedics’ (legal) protection and how to reword questions so that they would be closed-ended questions, such as those that asked a number, time, or period of symptom onset. In this instance, paramedics reflected on the essential information they needed to gather. For example, open questions such as “from what height did you/did the child fall from?” were changed to closed questions “did you fall from higher than 3 meters?” and “can you show me where the child fell from?”

In the workshops, we also worked on phrases for situations that paramedics perceived as challenging, for example dealing with aggressive or intoxicated people, cases of suspected domestic violence, an attempted or actual suicide, or when speaking to family members regarding the fatality of a relative.

Role play helped to establish whether the phrases covered all the important conceivable scenarios and identify unnecessary or unclear content.

Role plays revealed that nonverbal communication also plays an important role in encounters between paramedics and foreign-language patients. The observed nonverbal interaction was discussed with paramedics. As nonverbal communication may be culturally imprinted, the app also contains phrases for situations that one might think can be solved nonverbally. External medical professionals reviewed all phrases as to their accuracy. Before translation, the research team indicated potential ambiguities to prevent misleading translations. Also, all phrases were looked over by a cultural scientist to ensure comprehensibility and translatability. Some phrases though were reworded into simpler language or according to personal preference. In several cases, this changed the original intended meaning in a way that the phrase no longer fitted with (all) intended situations or did not reflect what was discussed with paramedics, resulting in the need for retranslation.

Subsequently, professional interpreters experienced in the medical field translated and audio-recorded the phrases. Translators were advised to use the most common and comprehensive wording. The results were then reviewed and partly retranslated to ensure high quality of the translations.

The app contains 600 standard phrases, though the tailoring of these for adult patients, pediatric patients, and specific third parties as well as to accommodate the patient’s gender, which is needed in many languages, meant that up to 1200 phrases were produced in Arabic, for example, and overall, 16,000 audio-files were generated in total (the app is available for Android and iOS, see Multimedia Appendix 2).

### Structure

We faced the challenge of grouping the comprehensive content in a way that all phrases could be easily found. Different grouping options, for example according to organ systems, body regions, or potential patient outcomes, were discussed and tested. We also considered fixed series of phrases for certain recurring situations, such as a suspected acute coronary syndrome. A series approach was rejected since the content and course conversations can vary considerably from case to case.

The final adopted categorization approach arose from consideration of a combination of symptoms, on-the-spot (suspected) diagnoses, and incidents, sorted by probability of occurrence [25,26]. There are separate categories for physical examination, informative and reassuring sentences, questions concerning drugs, intolerances, preexisting conditions, and patient documents. Phrases that are necessary for the primary survey following the ABCDE (Airway, Breathing, Circulation, Disability, Exposure) approach form the first group. This systematic approach is recommended by the European Resuscitation Council, is a widely accepted standard of care for the immediate assessment and treatment of life-threatening clinical problems [27], and is applicable in all clinical emergencies.

The second group assembles questions that help uncover the reasons for seeking medical emergency care if not obvious or not known to the paramedics. Phrases may appear in more than one category, and categories are linked with each other for quick navigation. Within categories, the content is clustered according to the paramedic approach of structuring a rescue mission.

### Functions and Navigation

In the second phase of the app development process, the focus shifted toward the process of designing a user interface that reflected the functions that paramedics deemed helpful and allowed easy, rapid, and flexible navigation. In the following sections, we describe the additional functionality requested by the paramedics and how we implemented it.

#### Audio Playback and Additional Text Display

All phrases can be both played back audibly or displayed as text. The text is displayed in horizontal format, which facilitates reading for the patient. For languages that do not use the Latin alphabet, the correct reading direction is indicated by an arrow.

#### Log to Document Patient Responses

Paramedics indicated that they would like to document the patient responses and to review them at any time. Two possible log views were identified as the most convenient. First, an overview of the documented answers (“yes,” “no,” or “unclear” for closed-ended questions; localization of pain or an injury marked on a ﬁgure) is provided using the SAMPLE history scheme (questions for a secondary assessment used in prehospital emergency care including Symptoms, Allergies, Medications, Past medical history, Last oral intake, Events prior to incident) [28]. This function additionally allows paramedics to quickly identify if certain information has not yet been obtained, which therefore facilitates a complete assessment. Second and alternatively, a “chat view” shows the complete course of conversations, including information that was provided.

#### Quick Access Menu

Phrases that are expected to be used frequently are grouped, and these can be accessed by selecting a steady button that serves as a “quick access menu.” We have not implemented the possibility to customize this function because the app is to be installed on a jointly used device on the rescue vehicles.

#### Navigation

**Figure 4:**
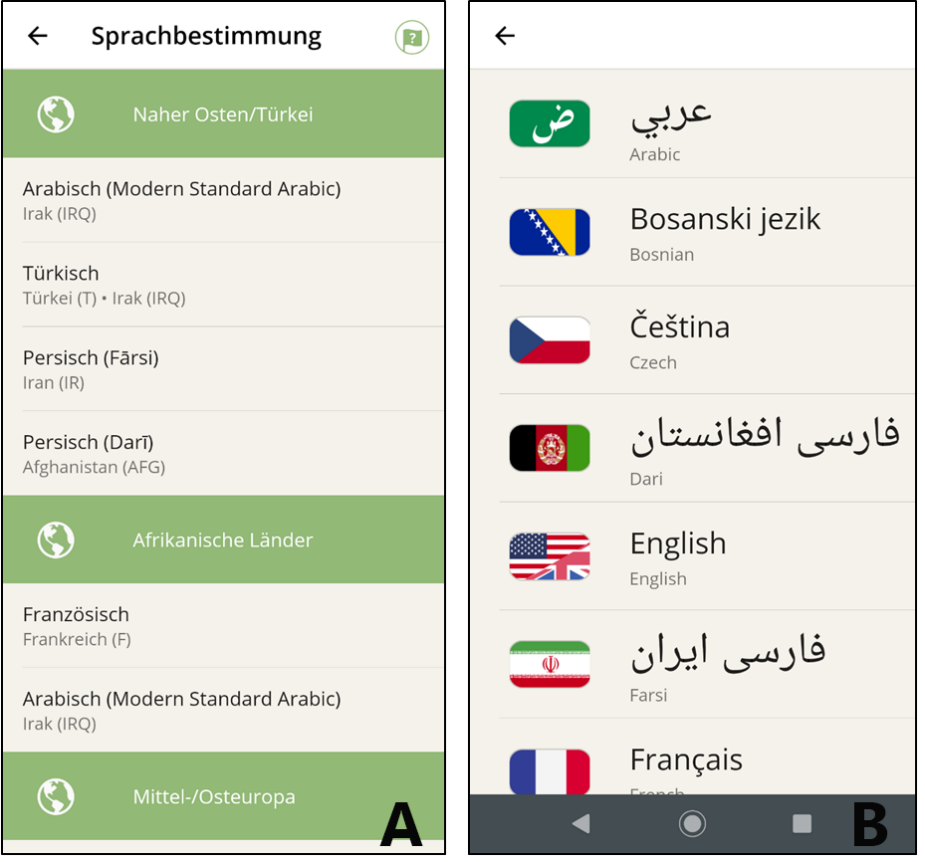
Paramedics select the language from (A) a geographically sorted directory or (B) have the patient choose from a flag button list that is sorted alphabetically and labeled in the original language.

When starting the app, paramedics have to choose age (adult or child) and gender of the person in need of help. Based on this selection, the app automatically adopts age- and sex-speciﬁc content and wording to address children and their health problems, for example. Paramedics then select the language the patient (presumably) speaks from a geographically sorted directory, or the patient selects a language they understand from a flag button list (Figure 4).

Other options, such as trying to ask the patient to show his or her home country on a map, were discarded as some patients may not be able read a map, while others might be unsure whether the paramedics are asking for their country of birth or where they have travelled from. Additionally, in many countries, more languages and dialects are spoken and cannot be identified with certainty using maps.

**Figure 5:**
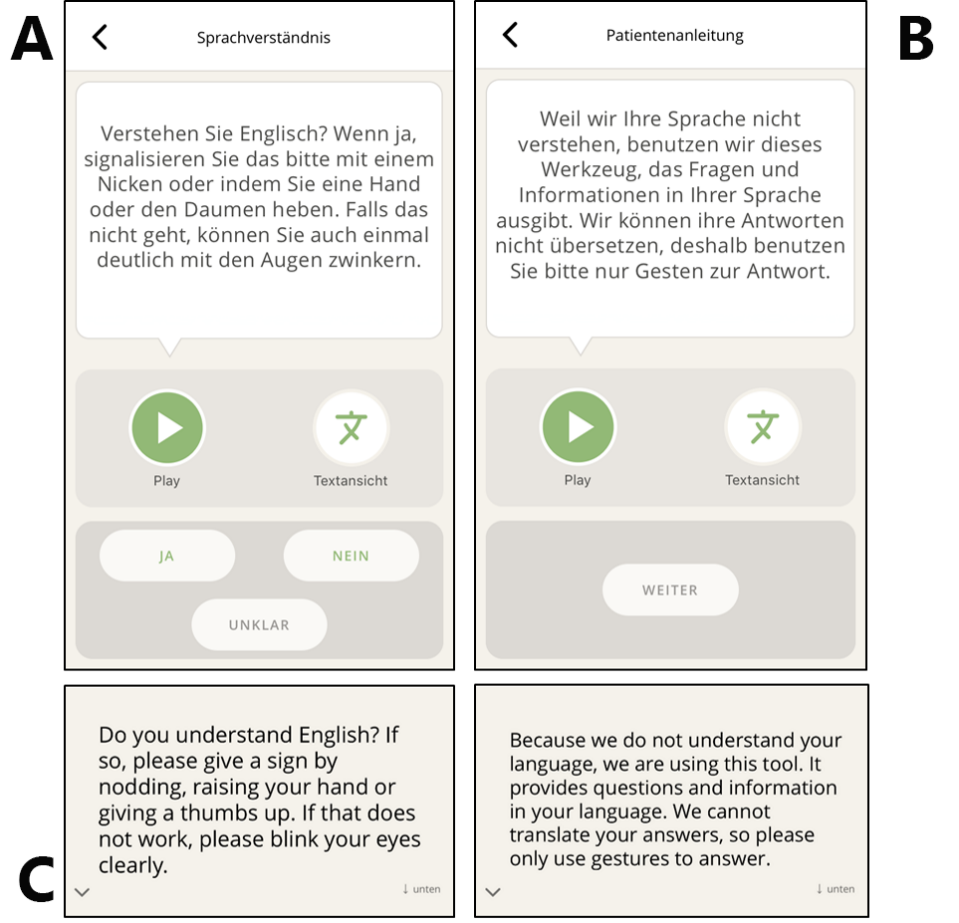
(A) To confirm that the patient understands the chosen language, the app may ask “Do you understand [chosen language]?” and asks the patient to sign “yes” if so. (B) The app provides a short explanation of the tool’s function to prevent misunderstandings. (C) The respective text is displayed using the “Textansicht” button.

A patient’s comprehension of the language selected may be checked by asking the patient to give a sign. The used phrase considers that affirmative and rejecting gestures are not universal across cultures and languages. Then, the use of the app is briefly explained to the patients (Figure 5). If required, the language check and app explanations may be skipped to reach the category list more quickly.

The app navigation takes into account the variety and complexity of rescue missions, for example by allowing the user to switch quickly between addressees (ie, patient or accompanying companions) to reach the patient response log at any time or to change directly to a related category without navigating through category lists again.

In early versions of the app, paramedics experienced difficulties in locating the function to change the addressees and the output language. To address this, icons were redesigned and more appropriately arranged.

### Device Requirements

Emergency medicine places high demands on the used devices: They have to be resistant to falls, dust, water splashes, and extreme temperatures as well as easy to disinfect and portable so that they are easily carried in rescue missions. They need a long-lasting battery, the displayed content must be legible in sunlight, and the audio output has to be loud enough for noisy situations or to address hearing impaired patients. Different smartphones and tablets and accessories were discussed and tested with the paramedics and the software designers.

For the study, we decided to use a Motorola Play Z2 Android smartphone with an attachable extra speaker, dedicated battery, a sound output of up to 84 decibels, a turbo power charger, a scratch-and crack-resistant glass cover, and a shock-absorbing bumper.

### Languages and Dialects

The selection of languages that needed to be supported by the study version of the app was based on the questionnaires to paramedics regarding recent rescue missions with foreign-language patients, demographic statistics, and cross-border travel data. For this study, the app supports 18 languages and dialects: Arabic, Bosnian, Croatian, Czech, Dari (Persian), English, Farsi (Persian), French, German, Italian, Kurdish-Sorani, Lithuanian, Pashto (Afghani), Polish, Russian, Serbian, Spanish, and Turkish.

### Experience Obtained From the Development Process

We used a variety of techniques to explore obstacles faced by paramedics in rescue missions with foreign-language patients and to devise an app to tackle these. Our approach allowed both researchers and software designers to understand the paramedics’ daily work, perspectives, priorities, and experienced problems as well as their needs and expectations towards an intervention aimed at overcoming language barriers. Paramedics discussed phrases, app structure, and scenarios and tested paper prototypes, click dummies, and preliminary app versions. Their engagement as well as our field observations allowed us to reveal and subsequently consider paramedic experience-based knowledge, and both were invaluable to designing and refining the app. We would not have gained this insight relying on literature and guidelines alone. For example, parents of sick toddlers must be asked whether they have a baby carrier to guarantee safe transport.

The engagement of paramedics with different professional experiences, ages, genders, and attitudes towards (digital) innovations was also valuable. In heterogeneous groups, we observed some controversial and inspiring discussions. For instance, paramedics in their early career expressed in the workshops that they adhered much more to guidelines (eg, they always followed the ABCDE scheme). In contrast, more experienced paramedics barely relied on these, which we also observed in role plays and during field observations. As our app needed to reflect the different ways paramedic users think and act, discussions helped to reconcile these different views.

The simulation of different rescue scenarios with both the paper prototypes and preliminary app versions helped us to check whether the features and content discussed in theory were of value and really needed in practice. The tests to explore how paramedics navigate through the app helped to build a user-friendly app.

The decisions regarding techniques used to explore the needs of paramedics were partly based on previous experience and on new questions and challenges that arose during the research and development process.

This open approach was especially helpful as it became clear that emergency medical services is an area that is also characterized by tacit knowledge and experience that is difficult to verbalize. Additionally, we discovered that the paramedics themselves often had different approaches when engaged in developing and testing activities. Some paramedics found it easier to reflect on personally experienced rescue missions with foreign-language patients and to assess their own behavior in these situations. For others, role play turned out to be a very effective tool as the scenarios helped paramedics imagine a concrete situation and this approach also involved more reserved participants. Paramedics became their own role model, and in some simulations, they acted contrary to their personal expectations. Through their involvement, they discovered processes or behavioral patterns they were previously unaware of, for example gestures used automatically to enhance communication. Role play allowed both the paramedics and researchers to discover the paramedics’ tacit knowledge of their work. In discussions subsequent to role play, paramedics would reflect on the communication process and combine their opinions in a very productive way.

The paramedics that tested the final app preferred to report their experience with it directly to our study nurse rather than using the messenger channel. These paramedics were familiar with the functionality and the content of the app and also provided peer teaching in the first phase of implementation.

## Discussion

### Principal Findings

In an action-oriented participatory approach, we successfully developed a digital communication tool to overcome language barriers in prehospital emergency care together with the end users — paramedics from 4 rescue stations. As the software designers took part in this process from the beginning, they gained background knowledge and were able to understand and discuss paramedics’ needs and expectations regarding the functions requested. Pre-assumptions were challenged, which led to a greater understanding of the field. Communications and collaboration are challenging, especially if perspectives are contrary or if requirements cannot be met. Still, this complex approach ensured that the communication tool met the paramedics’ needs, as it considered their perspectives informed by the problems they experienced in their day-to-day work. This is likely to increase the success of implementation in the longer term. In contrast to existing translation tools such as Google Translate, the developed app accommodates the needs of rescue service operations and enables quick and easy handling. It is independent of cell phone network coverage, and the translations were subject to an extensive quality assurance process. The possibility of documenting the communication process and patient responses are further aspects not offered by other solutions to date. Additionally, the app complies with current data protection and security standards.

Over the course of this study, it became apparent that a by-product of the app development was that it raised awareness among the participating paramedics of the communication barriers with foreign-language patients and the effect this has on the provision of care. It made paramedics think about their role and their previous behavior and attitudes, and they discussed these issues with their colleagues.

### Recommendations

This experience is captured in the following sections, and from it emerges a set of recommendations for future medical software development.

#### End Users Are Experts of Their Work Environment

Paramedics generated ideas concerning the app content and structure, user interface, and technical requirements. This ongoing user input in iterative cycles helped researchers and the software designers understand paramedics’ needs. We experienced that end users were able to reflect on highly complex issues. Thus, end users need to be taken seriously as experts of their work environment. In line with previous research [22,29], we argue that any new digital interventions in health care should actively involve end users in the design process, and we additionally recommend engaging software developers from the earliest stages of the project.

#### A Participatory Approach Requires Time, Patience, and Humility

During such an open process, unexpected issues might and can be expected to arise. It is essential to try not to anticipate the results, but to be open minded to any issues that come up. Planning a sufficiently long period for user testing of mock-ups facilitates input, feedback, and time to make specific adjustments prior to implementation.

#### A Complex Intervention Benefits From a Range of Development Techniques

The simulation of rescue scenarios with prototypes and other mock-ups was very helpful to structure and improve the content. Paramedic feedback on the user interface of the early app versions as well as the “think-aloud tasks” helped to build a user-friendly app. Therefore, we would encourage researchers to try a variety of approaches to foster development activities.

#### The Development of a Digital Intervention for Health Care Requires a Multidisciplinary Team

Working in a multidisciplinary team of clinical scientists, software developers, designers, and cultural scientists was crucial to meeting the challenge of building a communication tool suitable for paramedics in emergency medical services. We therefore recommend establishing a multidisciplinary team, ensuring that both clinical and technical competences are present.

#### Sincerity is the Best Policy

We were unable to fulfil all the needs identified during the development process. However, as we discussed, conflicting views on functionality with paramedics and explaining why we could not include certain functions did not diminish but rather enhanced their commitment. Should this be due to a lack of resources, we suggest having users prioritize their ideas and consider whether to incorporate disregarded functions in future versions. We also recommend discussing openly why some user-requested functions are not incorporated.

#### Translation Poses the Risk of Altering the Intended Concepts and Meanings to Be Relayed

Even though potential ambiguities were meant to be indicated in the final version of the phrases, some translations were found to be unintentionally misleading. We recommend reviewing all phrases before and after translations with a person(s) proficient in the respective languages and medical professionals. Various rounds of forward translation or forward- and backward translation increase the validity of the translation and reduce the risk of mistranslation [30].

#### Anticipate Future Ways to Use the New Technology

The software developers contributed valuable ideas regarding further opportunities and challenges that may arise after app deployment. For example, they considered possibilities to directly transfer the documented data into electronic health records. The app was optimized so it could be run on different devices (eg, tablets) and different operating systems, to ease adoption and dissemination. In order to minimize potential compatibility issues, we recommend thinking about potential future use at an early stage and to attempt to accommodate this, where it is possible to do so, but not necessarily including it functionally.

### Conclusions

Clinician researchers, paramedics, software designers, and translators alone could not have built and created this comprehensive app. The expertise of this multiprofessional team was key to the success of developing this app. Digitization in today’s health care needs creative alliances between practitioners and developers: Bringing end users and software designers together, encouraging them to discuss ideas and opinions, giving a voice (and a say) to end users, and acknowledging their views on content and functionality are vital for developing digital innovations in health care.

### Outlook

Currently, the app is utilized in the work routines in the participating rescue stations. Within an interventional trial, we will assess the app’s usability, acceptance, effect on communication with foreign-language patients, and effect on information collection. We hope that our tool contributes to better and safer provision of paramedic care for foreign-language patients. We also plan to have the app evaluated and discussed by patient representatives with language skills in the supported languages.

Due to the COVID-19 pandemic, we upgraded the app with COVID-19–relevant questions covering associated symptoms and important information as well as phrases to communicate self-protection measures. The marketed version of the app has been released and is available for Android and iOS as “aidminutes.rescue (COVID-19)” in various app stores.

## Appendix

Multimedia Appendix 1 Metamorphosis of app development and impressions of the participative development techniques. A-D Metamorphosis of app development with phrase construction and grouping (A), paper-based app (B), first click dummies (C) and final app (English interface) (D). E-H is showing participative approaches with workshops with paramedics in the rescue stations (E, F), and case simulations with paper-based app (G) and preliminary versions (H).

Multimedia Appendix 2 Links to the app stores (Android and iOS).
