# Supplementary figures and images for: Designing an App to Overcome Language Barriers in the Delivery of Emergency Medical Services: Participatory Development Process
Source: JMIR Mhealth Uhealth. 2021 Apr 14;9(4):e21586. doi: 10.2196/21586 (PMC8082383; doi:10.2196/21586)

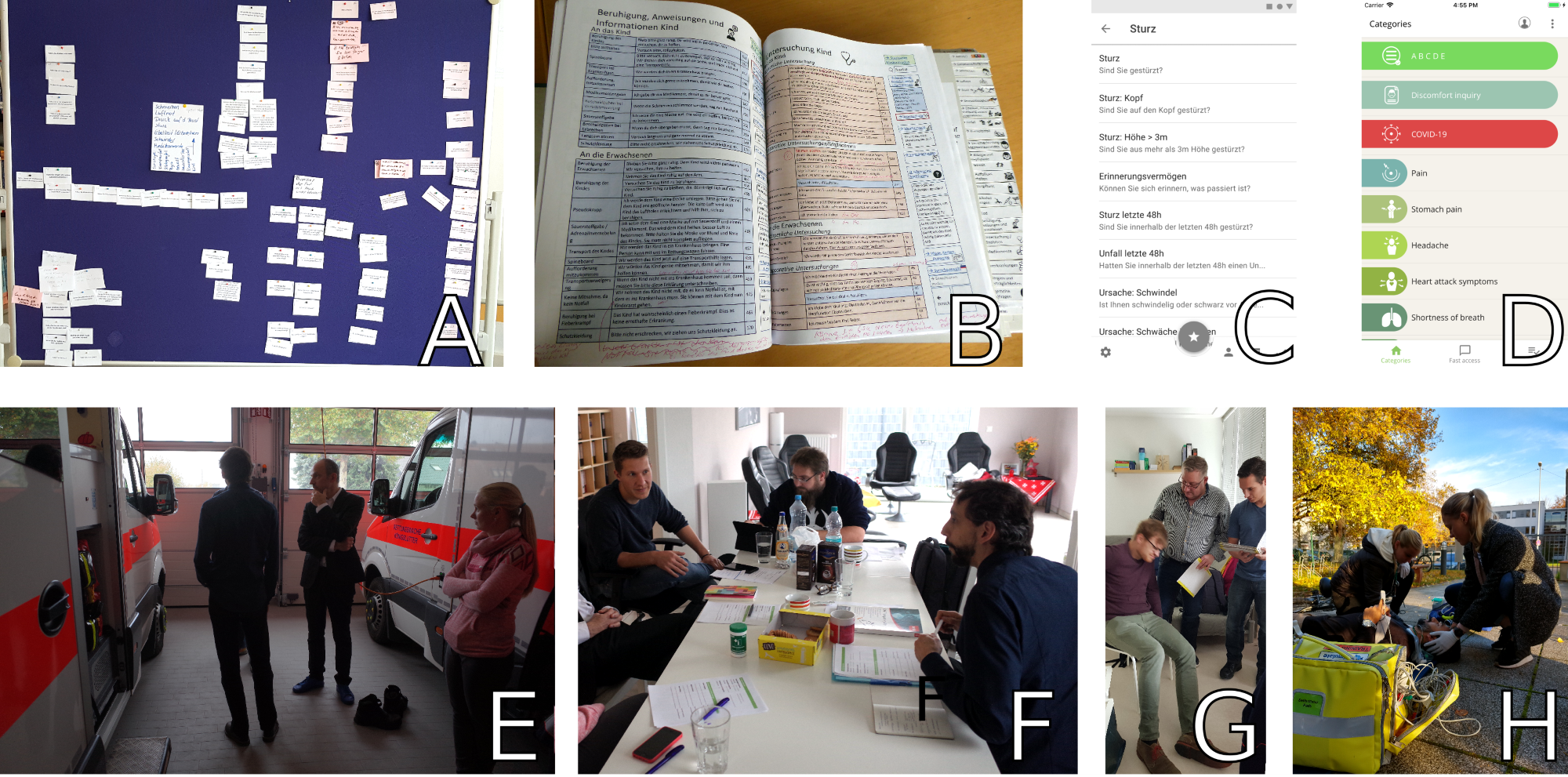

Supplement: Multimedia Appendix 1 [file mhealth_v9i4e21586_app1.png]
