# Supplementary material for: Designing an App to Overcome Language Barriers in the Delivery of Emergency Medical Services: Participatory Development Process
Source: JMIR Mhealth Uhealth. 2021 Apr 14;9(4):e21586. doi: 10.2196/21586 (PMC8082383; doi:10.2196/21586)
Supplement: Multimedia Appendix 2 [file mhealth_v9i4e21586_app2.docx]

**Google Play (Android)**


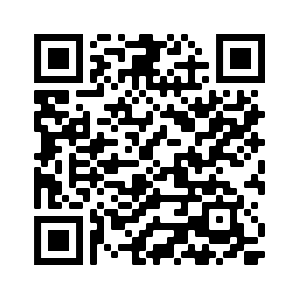


<https://play.google.com/store/apps/details?id=com.aidminutes.rescue.covid19>

**Apple Store (iOS)**


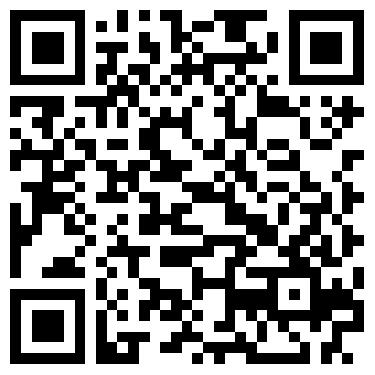


<https://apps.apple.com/app/aidminutes-rescue-covid-19/id1507581865>
